# Generalizing boundaries for triangular designs, and efficacy estimation at extended follow-ups

**DOI:** 10.1186/s13063-015-1018-1

**Published:** 2015-11-16

**Authors:** Annabel Allison, Tansy Edwards, Raymond Omollo, Fabiana Alves, Dominic Magirr, Neal D. E. Alexander

**Affiliations:** Lancaster University, Lancaster, LA1 4YW UK; The University of Sheffield, Western Bank, Sheffield, S10 2TN UK; MRC Tropical Epidemiology Group, London School of Hygiene and Tropical Medicine, Keppel Street, London, WC1E 7HT UK; Drugs for Neglected Diseases initiative (DNDi) Africa, Centre for Clinical Research, Kenya Medical Research Institute, Nairobi, Kenya; Maseno University, Private Bag, Maseno, Kenya; DNDi, Geneva, Switzerland; Centre for Medical Statistics, Medical University of Vienna, Spitalgasse 23, Vienna, 1090 Austria

**Keywords:** Randomized trials, Sequential methods, Triangular boundaries, Visceral leishmaniasis, Shrinkage estimator

## Abstract

**Background:**

Visceral leishmaniasis (VL) is a parasitic disease transmitted by sandflies and is fatal if left untreated. Phase II trials of new treatment regimens for VL are primarily carried out to evaluate safety and efficacy, while pharmacokinetic data are also important to inform future combination treatment regimens. The efficacy of VL treatments is evaluated at two time points, *initial cure*, when treatment is completed and *definitive cure*, commonly 6 months post end of treatment, to allow for slow response to treatment and detection of relapses.

This paper investigates a generalization of the triangular design to impose a minimum sample size for pharmacokinetic or other analyses, and methods to estimate efficacy at extended follow-up accounting for the sequential design and changes in cure status during extended follow-up.

**Methods:**

We provided R functions that generalize the triangular design to impose a minimum sample size before allowing stopping for efficacy. For estimation of efficacy at a second, extended, follow-up time, the performance of a shrinkage estimator (SHE), a probability tree estimator (PTE) and the maximum likelihood estimator (MLE) for estimation was assessed by simulation.

**Results:**

The SHE and PTE are viable approaches to estimate an extended follow-up although the SHE performed better than the PTE: the bias and root mean square error were lower and coverage probabilities higher.

**Conclusions:**

Generalization of the triangular design is simple to implement for adaptations to meet requirements for pharmacokinetic analyses. Using the simple MLE approach to estimate efficacy at extended follow-up will lead to biased results, generally over-estimating treatment success. The SHE is recommended in trials of two or more treatments. The PTE is an acceptable alternative for one-arm trials or where use of the SHE is not possible due to computational complexity.

**Trial registration:**

NCT01067443, February 2010.

**Electronic supplementary material:**

The online version of this article (doi:10.1186/s13063-015-1018-1) contains supplementary material, which is available to authorized users.

## Background

Visceral leishmaniasis (VL) is a parasitic disease transmitted by sandflies and is fatal if left untreated. It is estimated that there are 0.2–0.4 million incident cases per year, with the six worst affected countries—India, Bangladesh, Sudan, South Sudan, Ethiopia and Brazil—reporting more than 90 % of them [1].

Until recently, the first-line treatment for VL was daily injections with the antimonial compound sodium stibogluconate for 30 days. The current first-line treatment in eastern Africa is 17 days of daily injectable doses of sodium stibogluconate and paramomycin [2]. Sodium stibogluconate is associated with cardiotoxicity, and drug resistance is a concern in Asia [3]. From a public health perspective, safe and efficacious short-course combination therapies of less than 2 weeks duration with one or two oral treatments would be considered most desirable [3].

A number of trial designs have been adopted in the conduct of clinical trials against VL and other neglected tropical diseases, with the use of adaptive/sequential designs gaining prominence, particularly for Phase II trials. In particular, the triangular design allows for repeated interim analyses, each on a relatively small number of patients, to efficiently triage between poorly performing treatments and those showing promise for further investigation (e.g., inclusion in a Phase III trial) [4]. Effectively, the design acts as a decision tool for whether or not a treatment merits continued study. By pre-specifying the power, Type I error, and null and alternative values for the outcome (e.g., proportion cured), a closed continuation region is drawn with upper and lower boundary lines forming a triangular shape. If higher values are beneficial, then crossing the upper boundary corresponds to stopping for promise, while crossing the lower boundary corresponds to stopping for lack of promise. Variations on the design accommodate comparative trials [5].

The estimation of efficacy at the end of the trial using the full sample of data needs to account for the multiple interim analyses, which lead to bias in the maximum likelihood estimator (MLE), e.g., the simple proportion of the number of patients cured divided by the number of treated. One suitable option is the median unbiased estimate (Chapter 5 in [6]), which can be found using software such as R or SAS.

In VL treatment trials, there are two important efficacy endpoints: initial cure and definitive cure. After completion of treatment, initial cure status is established before the patient is discharged. In a clinical trial with a parasitological assessment conducted as standard at the end of treatment, a patient may have:

- cleared parasites (initial treatment success)
- parasites remaining but with clinical improvement, so that additional treatment is not considered to be required prior to discharge (potential slow responder to treatment)
- parasites remaining and clinical improvement not shown, and so they require rescue treatment prior to discharge (confirmed treatment failure)

**Fig. 1:**
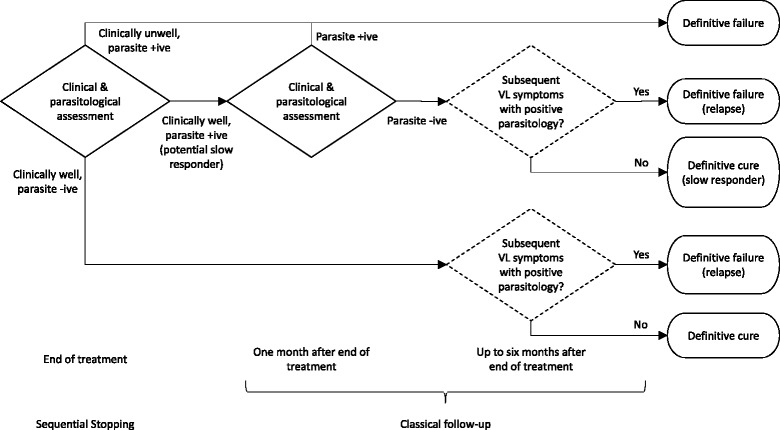
Flowchart outlining the assessments in a visceral leishmaniasis trial. A diagram showing the different clinical and parasitological assessment time points carried out and possible outcomes in a VL trial

For those not needing rescue treatment, definitive cure is assessed after a period of time (usually 6 months post end of treatment) due to the possibility of (1) slow response to treatment and (2) relapse following an initial treatment success. Slow response to treatment is confirmed with an additional clinical and parasitological assessment, usually 1 month after end of treatment. If parasites are cleared, the patient does not need rescue medication, this being a subset of treatment success at definitive cure. If parasites remain, the patient will have indication of rescue treatment, and will be considered a definitive failure at the 6 months’ follow-up. Future research direction decisions are based on definitive cure at the end of the follow-up period, as patient status may change between end of treatment and final definitive assessment. Figure 1 highlights the different assessment time points and possible outcomes.

In a Phase II randomized trial of VL treatments in Sudan and Kenya, a triangular continuation region was defined for each of the three arms [7]. In this trial, the primary endpoint, used for sequential decision-making, was initial cure: a binary outcome defined by absence or presence of parasites at end of treatment. This endpoint permitted the prompt identification of poorly performing treatments. Use of definitive cure in sequential decision-making was deemed not to be feasible due to implications for time to trial completion and the potential to expose an unacceptable number of patients to ineffective treatments.

The investigators found it necessary to modify the standard triangular design in two ways. Firstly, a pharmacokinetic (PK) evaluation component required a minimum of 30 patients, whereas the interim analysis was to be done after every 15 patients had completed the end-of-treatment assessment. So recruitment was to continue even if the upper boundary was crossed at the first interim analysis. Secondly, although standard sequential methods sufficed for the end-of-trial analysis of initial cure, a new methodology was needed for point and interval estimation for definitive cure, to account for sequential stopping, then non-sequential follow-up, operating in tandem.

The first modification ignored the impact on Type I and II errors, rather than attempting to adjust the standard continuation region. And the second modification consisted of a simple probability tree argument whose efficiency, in the statistical sense of low standard error, was not evaluated.

### Aims

Hence, in the current paper, we aim to derive methods for the following two distinct aspects of such trials:

- definition of flexible boundaries that generalize simple triangular ones to prevent stopping before a given number of patients, while maintaining nominal error probabilities
- estimation, by shrinkage and other methods, of the outcome variable (e.g., cure) at a time point later than that used for the sequential stopping rule

### Motivating example

In the motivating trial example [7], known as LEAP 0208, initial cure/end of treatment was at day 28 and definitive cure at day 210. The trial was non-comparative, with each arm having the same triangular boundary and being subject to the same stopping rules independently of the other two arms.

It was assumed that a day 28 cure rate of *p*_0_=0.75 could be achieved on standard care. Denoting the day 28 cure rate on treatment *j* by *p*_*j*_, the treatment effects were parameterized as

$$\theta_{j} = \log \left\lbrace \frac{p_{j}(1-p_{0})}{(1-p_{j})p_{0}} \right\rbrace, \quad \theta_{j} \in (-\infty, \infty), \quad j=1,2,3. $$

As a cure rate of 0.9 would be considered sufficiently promising to warrant further investigation, in this case

$$\theta_{\mathrm{R}} = \log \left\lbrace \frac{0.9(1-0.75)}{(1-0.9)0.75} \right\rbrace = 1.10. $$

The Type I and Type II error rates were both set at 5 %, so it was required that for each null hypothesis *H*_0*j*_: *θ*_*j*_=0, *j*=1,2,3:

(1) $$\begin{array}{*{20}l} P(\text{reject}~H_{0j}; \theta_{j} = 0) &= 0.05,  \end{array} $$

(2) $$\begin{array}{*{20}l} P(\text{reject}~H_{0j}; \theta_{j} = \theta_{\mathrm{R}}) &= 0.95.  \end{array} $$

Omollo et al. found the boundaries using analytical results specific to a triangular test, as described by Ranque et al. [8], rather than numerical integration. From the planned frequency of interim analyses—every 15 patients in each arm—and other parameters such as the error probabilities, equations for the upper and lower straight line boundaries are obtained. The maximum sample size is given by the intersection of these lines, in this case 63 per arm.

An additional complication in this trial was that at least 30 patients were required to assess PK. Therefore, although crossing the lower boundary at the first interim analysis would result in the arm being stopped for ethical reasons, crossing the upper boundary was to be disregarded, with recruitment continuing until the second interim analysis. This partial disregarding of the boundaries affects the stopping probabilities and hence, deviates the error probabilities from their nominal values. This was ignored in the trial described by Omollo et al., but such modified stopping rules are developed formally in the current paper, and we derive corresponding adjustments under asymmetric stopping or unequally spaced analyses.

## Methods

### The group-sequential triangular design

To review the triangular design briefly [6], consider a parameter of interest, *θ*, which could represent either the advantage of an experimental treatment over a control arm in a two-arm study, or relative to a fixed null value in a single-arm trial. The null hypothesis to be tested is *H*_0_: *θ*=0 against the alternative *H*_1_: *θ*>0, subject to error constraints:

(3) $$\begin{array}{*{20}l} P(\text{reject }H_{0}; \theta = 0) &= \alpha \end{array} $$

(4) $$\begin{array}{*{20}l}  P(\text{reject }H_{0}; \theta = \theta_{\mathrm{R}}) &= 1 - \beta \end{array} $$

for suitably small *α* and *β*, where *θ*_R_ represents the smallest possible treatment effect size that would be considered interesting or clinically relevant.

There are *k* looks at the data, with the data available at the *i*th interim analysis summarized in terms of the efficient score for *θ*, denoted by *B*_*i*_, and Fisher’s information, denoted by *V*_*i*_ (see p. 107 [9]). For most types of data, there is a straightforward relationship between Fisher’s information and the sample size, *n*_*i*_. For example, for a single stream of binary data *V*_*i*_=*n*_*i*_*p*(1−*p*), where *p* is the success probability [6]. Two key assumptions underpinning most sequential methods are that $B_{i} \sim \mathcal {N}(\theta V_{i}, V_{i})$, and that (*B*_*i*+1_−*B*_*i*_) and *B*_*i*_ are independent, *i*=1,2,…. The standardized test statistic is denoted by $Z_{i} = B_{i}/\sqrt {V_{i}}$ and follows the standard normal distribution when *θ*=0 [10]. These assumptions have been shown to be valid for a wide variety of response types [6].

The efficient score is compared to a set of upper and lower boundary values at the *i*th analysis, *i*=1,…,*k*. At the first (*k*−1) interim analyses, if $B_{i} \geq u_{i}\sqrt {V_{i}}$, the trial is stopped and the null hypothesis rejected; if $B_{i} \leq \ell _{i}\sqrt {V_{i}}$, the trial is stopped and the null hypothesis not rejected, and the trial is continued otherwise. We reject the null hypothesis at the final analysis if $B_{k} \geq u_{k}\sqrt {V_{k}}$, and fail to do so otherwise as we define *ℓ*_*k*_=*u*_*k*_.

**Fig. 2:**
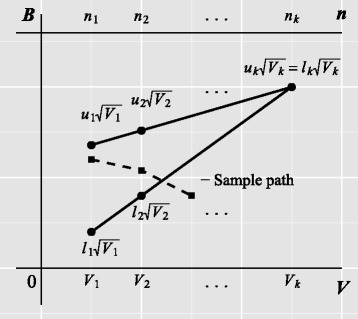
Illustration of the triangular group-sequential design. Boundaries for plotting the efficient score (*B*_*i*_) versus Fisher’s information (*V*_*i*_) for *k* analyses. The sample path shows one possible route the analysis could take. Here, the trial would be stopped for inefficacy at the third interim analysis

To design a group-sequential trial, we need to choose 3*k*−1 values (*V*_*i*_,*ℓ*_*i*_ and *u*_*i*_, with the subscript for *ℓ* running only to *k*−1, since *ℓ*_*k*_=*u*_*k*_), such that (3) and (4) are satisfied. It is clear that additional constraints are required to produce a unique trial design. This involves specifying the spacing of the information levels, i.e., specifying constants *r*_2_,…,*r*_*k*_ and imposing that *V*_*i*_=*r*_*i*_*V*_1_ for *i*=2,…,*k*. Additionally, formulae for the boundary values, *l*_*i*_=*l*_*i*_(*a*) and *u*_*i*_=*u*_*i*_(*a*), are provided in terms of a single common parameter, *a*. This leaves just two unknowns, *a* and *V*_1_, to be found by solving (3) and (4). For example, to create triangular boundaries,

(5) $$\begin{array}{*{20}l} \ell_{i} &= -a\{1 - 3(r_{i}/r_{k})\}/\sqrt{r_{i}}  \end{array} $$

(6) $$\begin{array}{*{20}l} u_{i} &= a\{1 + (r_{i}/r_{k})\}/\sqrt{r_{i}}  \end{array} $$

for *i*=1,…,*k* [11]. A SAS implementation is available [11]. At each analysis, *B*_*i*_ is compared to $\ell _{i}^{*} = \ell _{i} \sqrt {V_{i}}$ and $u_{i}^{*} = u_{i} \sqrt {V_{i}}$. The triangular shape can be seen on a plot of *B* against *V* (Fig. 2).

Note that if for any reason one wishes to avoid stopping early for efficacy at analysis *i*, for some *i*<*k*, then it is straightforward to impose *u*_*i*_:=*∞* before solving for *a* and *V*_1_.

### Fixing an interim analysis after a pre-specified number of responses

Whilst phase II trials primarily look at the safety and efficacy of a treatment, they may also be concerned with the PK properties of a new treatment, and a specific number of participants will be required for this. Here, we discuss how to design a group-sequential trial where the first interim analysis occurs after a pre-specified number of observations, *n*_1_=*n*^′^, or, equivalently, a fixed first information level, *V*_1_=*V*^′^.

Since we can no longer fix *V*_*i*_=*r*_*i*_*V*_1_ for *i*=2,…,*k* (as this would arbitrarily fix the total sample size), we instead specify *r*_3_,…,*r*_*k*_ and require *V*_*i*_−*V*_1_=*r*_*i*_(*V*_2_−*V*_1_) for *i*=3,…,*k*. The boundary Eqs. (5) and (6) remain unchanged.

In principle, this is a very straightforward modification, and one must again solve two equations for two unknowns. However, the solution becomes slightly more difficult due to the effect of this modification on the correlation structure of the interim test statistics. In the original problem, one can simplify the computation because the correlation structure depends only on the relative spacing of interim analyses and not on the absolute sample sizes. Specifically, setting *V*_*i*_=*r*_*i*_*V*_1_, *l*_*i*_=*l*_*i*_(*a*) and *u*_*i*_=*u*_*i*_(*a*) for *i*=1,…,*k*, one can first solve Eq. (1) for *a* via a one-dimensional search or root-finding routine and subsequently solve Eq. (2) for *V*_1_. If, however, we now fix *V*_1_, then the relative spacing and, consequently, the correlation structure, is dependent on the absolute total sample size. Thus, (1) and (2) can only be solved by a two-dimensional search or root-finding routine. As this option is not available in standard software for designing group-sequential trials, we have written R functions and made them available here as supplementary material (see Additional file 1). Note that the same idea can be used to pre-specify the information levels at more than one interim analysis.

We considered three potential designs for the example in ‘Motivating example’. In Design 1, two interim analyses are planned, with the first occurring after a fixed 30 observations. Design 2 is similar, except that an additional earlier interim analysis is planned after 15 responses, with no early stopping for efficacy (*u*_1_=*∞*). In Design 3, six interim analyses are planned, where the first three are fixed at 10, 20 and 30 observations, with the first two analyses not allowing early stopping for efficacy (*u*_1_= *u*_2_=*∞*).

We produced a graph of the boundaries for plotting the efficient score (*B*_*i*_) versus Fisher’s information (*V*_*i*_), and for each design, investigated the operating characteristics by simulating 10,000 experiments under both the null and alternative hypotheses. Specifically, the Type I error, power and expected sample size were calculated.

### Estimation of the probability of cure after extended follow-up

Consider estimating the probability of cure after extended follow-up (day 210) in the motivating example. For treatment *j*=1,2,3, the MLE is

$$\hat{p}_{210, j} = S_{210,j} / n_{210,j}, $$

where *S*_210,*j*_ denotes the number of successes (patients cured) at day 210. If we focus on the treatment *j*^∗^ providing the apparent largest effect size, i.e.,

$$j^{*} = \arg \max_{j = 1,2,3}\hat{p}_{210, j}, $$

then $\hat {p}_{210, j^{*}}$ is a biased estimate of $p_{210, j^{*}\phantom {\dot {i}\!}}$ for two reasons. Firstly, due to the potential early stopping of the trial based on the day 28 endpoint. Note that

(7) $$ \hat{p}_{210, j} = \hat{p}_{j}\hat{q}_{j} + (1 - \hat{p}_{j})\hat{s}_{j}  $$

where $\hat {p}_{j}$ denotes the day 28 success proportion, $\hat {q}_{j}$ denotes the proportion of patients who remain cured at day 210 (out of those already cured at day 28) and $\hat {s}_{j}$ denotes the proportion of patients who switch from not cured at day 28 to cured by day 210 (out of those not cured at day 28, i.e., slow responders). It is well known that $\hat {p}_{j}$ tends to overestimate *p*_*j*_ when a trial is stopped early for efficacy (and underestimate *p*_*j*_ when a trial is stopped early for futility) [6], and this bias will be inherited by $\hat {p}_{210, j}$. The second source of bias is due to the multiple treatments being evaluated. Assuming that the success probabilities of the three treatments are reasonably similar, it is intuitively clear that if we focus our attention on the largest success proportion, then this proportion will tend to be an overestimate.

We now investigate two methods that aim to improve estimation compared to the MLE. The first is a probability tree estimator (PTE), which attempts to address the early stopping bias by replacing $\hat {p}_{j}$ in (7) with a median unbiased estimate. The second is a shrinkage estimator (SHE), which should help counteract both sources of bias by shrinking the three maximum likelihood estimates towards each other [12].

#### Probability tree estimator

In this PTE section, we omit the *j* subscript to simplify the notation. Estimation of the success probability in the initial period, *p*, is subject to sequential stopping, while the subsequent follow-up is not. Hence, Omollo et al. proposed estimating the proportion with definitive cure—denoted by *p*_210_—using a probability tree argument to separate the two periods [7]. More specifically, *p*_210_ is the sum of the probabilities of two events: (1) initial cure followed by cure at extended follow-up and (2) initial failure followed by cure during the extended follow-up. These probabilities are denoted *pq* and (1−*p*)*s*, respectively [*pr* and (1−*p*)*s* in the notation of Omollo et al.], i.e., *q* denotes the conditional probability of cure at day 210 given cure at day 28, and *s* denotes the conditional probability of cure at day 210 given no cure at day 28. Omollo et al. [7] proposed the following estimate for *p*_210_:

$$\tilde{p}_{210} = \tilde{p}\hat{q} + (1-\tilde{p})\hat{s}, $$

where $\tilde {p}$ is the median unbiased estimate of *p* [6], with $\hat {q}$ and $\hat {s}$ being estimated by maximum likelihood from the 2×2 table of cure status at the two time points. The first term, $\tilde {p}\hat {q}$, takes into account those patients who relapse—important in VL trials. Here, $\hat {q}$ is the proportion of patients cured after treatment (at day 28 in Omollo et al.’s trial) who do not relapse. The second term, $(1-\tilde {p})\hat {s}$, accounts for slow responders—those who are not cured initially (at day 28) but become so by follow-up (day 210). Here, $\hat {s}$ is the proportion of slow responders out of those patients who are not cured by day 28.

The sampling variance of this estimator was not derived by Omollo et al., so we do so here. We have

$$\text{Var}[\tilde{p}_{210}] = \text{Var}[\tilde{p}\hat{q}] + \text{Var}[(1-\tilde{p})\hat{s}] + 2\text{Cov}[\tilde{p}\hat{q}, (1-\tilde{p})\hat{s}]. $$

The first two terms in the above expression can be analyzed into variances of single parameters by using the standard formula for the variance of a product. We assume $\tilde {p}$, $\hat {q}$ and $\hat {s}$ are mutually independent. Denoting $\text {Var}[\tilde {p}]$ by *σ*^2^, and with an obvious notation for the variances of $\hat {q}$ and $\hat {s}$ then, for example, the first term becomes

$$\mathrm{E}(\,\tilde{p})^{2}{\sigma^{2}_{q}} + \mathrm{E}(\hat{q})^{2}\sigma^{2} + {\sigma^{2}_{q}}\sigma^{2}. $$

For the third term, some expectation parameters can be taken into brackets:

$$\begin{array}{*{20}l} \!\!\!\!\!\!\!\!\!2\text{Cov} [\tilde{p}\hat{q}, (1-\tilde{p})\hat{s}] & \,=\, 2\mathrm{E}[\tilde{p}\hat{q}(1-\tilde{p})\hat{s}]\! - 2\mathrm{E}[\tilde{p}\hat{q}]\mathrm{E}[\!(1-\tilde{p})\hat{s}] \\ &\!= 2\mathrm{E}[\hat{q}]\mathrm{E}[\hat{s}]\{\mathrm{E}[\tilde{p}(1-\tilde{p})]\\ &\quad- \mathrm{E}[\tilde{p}]\mathrm{E}[(1-\tilde{p})]\} \\ & \!= -2\mathrm{E}[\hat{q}]\mathrm{E}[\hat{s}]\{\mathrm{E}[\tilde{p}^{2}] - (\mathrm{E}[\tilde{p}])^{2}\}\\ & \!= -2\mathrm{E}[\hat{q}]\mathrm{E}[\hat{s}]\sigma^{2}. \end{array} $$

Bringing the terms together, replacing the expectation by the observed values of $\tilde {p}$, $\hat {q}$ and $\hat {s}$, and gathering by ${\sigma ^{2}_{q}}$ and ${\sigma ^{2}_{s}}$ yields:

$$\text{Var}[\tilde{p}_{210}] = {\sigma^{2}_{q}}\left(\,\tilde{p}^{2}+\sigma^{2}\right) + {\sigma^{2}_{s}}\left(\!\left(1-\tilde{p}\right)^{2}+\sigma^{2}\!\right) + \sigma^{2}(\hat{q}-\hat{s})^{2}. $$

A 95 % confidence interval (CI) can then be calculated using

$$\tilde{p}_{210} \pm z_{1 - \alpha/2} \sqrt{\text{Var} \left[\,\tilde{p}_{210}\right]} $$

where *z*_1−*α*/2_=1.96 is the 1−*α*/2 percentile of the standard normal distribution.

#### Shrinkage estimator

Shrinkage methods have a long and rich history in statistics, going back to the famous James–Stein estimator [13]. Their usefulness in the context of a clinical trial with multiple treatment arms and interim analyses has been shown by Carreras and Brannath [12]. See also [14].

In very general terms, the idea is that the estimate of treatment effect in one particular group borrows information about the treatment effect in all other groups [15]. More specifically in our context, consider a probit transformation of the (day 210) success probabilities,

$$\theta_{j} = \Phi^{-1}(\,p_{210,j}) \text{ for} j=1,2,3, $$

where *Φ* denotes the standard normal distribution function. We now make the assumption that the success probabilities are similar to each other, in the sense that they have a common prior distribution that is Gaussian with mean *μ* and variance *τ*^2^:

$$\theta_{j} \mid \mu, \tau^{2} \sim \mathcal{N}(\mu, \tau^{2}), \quad j = 1, 2, 3. $$

This produces the effect that, compared to the maximum likelihood estimate $\hat {\theta }_{j} = \Phi ^{-1}(\,\hat {p}_{210,j})$, the posterior mean of *θ*_*j*_ is shrunk towards *μ*—where the smaller *τ*^2^ is, the greater the amount of shrinkage. In reality, we treat *μ* and *τ*^2^ as unknowns and give them prior distributions. This produces the effect that the posterior means are shrunk towards *each other*, where the degree of shrinkage depends on how spread out the maximum likelihood estimates are. Our full model is

(8) $$\begin{array}{*{20}l} P(Y_{i,j} = 1) & = p_{210,j}, && i = 1, \ldots, n_{j}; j = 1, 2, 3,\\ p_{210,j} & = \Phi(\theta_{j}), && j = 1, 2, 3,\\ \theta_{j}|\mu, \tau^{2} & \sim \mathcal{N}(\mu, \tau^{2}), && j = 1, 2, 3,\\ \mu & \propto 1, \\ \tau^{2} & \sim \mathcal{IG}(\alpha, \beta), \\ \alpha & = 2, \\ \beta & = 0.3,  \end{array} $$

where *Y*_*i*,*j*_ is the day 210 response of the *i*th patient on treatment *j*, *Φ* is the cumulative distribution function of the standard normal distribution and $\mathcal {IG}$ denotes the inverse-gamma distribution.

Our prior distribution for *μ* is non-informative. However, we do not choose a non-informative prior for *τ*, as this leads to problematic inferences when the number of groups is small [16]. Instead, we use an inverse-gamma distribution for *τ*^2^. The model (8) can then be fitted using a relatively simple Markov chain Monte Carlo (MCMC) algorithm [17], and we provide R code in the supplementary material (see Additional file 1). The parameters of the inverse-gamma distribution are chosen based on our prior knowledge that a range of treatment effect sizes (*θ*_*j*_’s) of *Φ*^−1^(0.9)−*Φ*^−1^(0.75)≈0.6 was very plausible, but a range of effect sizes twice this large was fairly unlikely. For a normal distribution with variance 0.3, 42 % of the probability density lies within 0.3 of the mean, and 73 % of the density lies within 0.6 of the mean. It, therefore, seemed a reasonable and pragmatic choice to give *τ*^2^ a prior mean of 0.3. This implies that *β*=0.3(*α*−1). If *α* is chosen too large, the degree of shrinkage or borrowing is effectively fixed in advance, i.e., it does not depend on how spread out the treatment effect estimates are. On the other hand, if *α* is too small, the MCMC will run into convergence issues. We, therefore, found *α*=2 via a process of trial and error, such that the model gave acceptable performance in a range of simulated scenarios (see below).

The output of the MCMC algorithm is a sequence of draws $\theta _{j}^{(1)},\ldots,\theta _{j}^{(N)}$ from the posterior distribution of *θ*_*j*_ for *j*=1,2,3. To produce a point estimate for *p*_210,*j*_, we take the mean of the back-transformed draws $\Phi \left (\theta _{j}^{(1)}\right),\ldots,\Phi \left (\theta _{j}^{(N)}\right)$. A 95 % credible interval is found via the sample 2.5 *%* and 97.5 *%* quantiles of $\Phi \left (\theta _{j}^{(1)}\right),\ldots,\Phi \left (\theta _{j}^{(N)}\right)$.

#### Maximum likelihood estimator

As described previously, the MLE for the probability of cure after extended follow-up (day 210) in the motivating example is given by

$$\hat{p}_{210, j} = S_{210,j} / n_{210,j}, ~~~ j = 1, 2, 3, $$

where *S*_210,*j*_ denotes the number of successes (patients cured) and *n*_210,*j*_ denotes the number of patients at day 210. A 95 % CI is then given by

$$\hat{p}_{210, j} \pm z_{1-\alpha/2} \sqrt{\frac{\hat{p}_{210, j}(1 - \hat{p}_{210, j})}{n_{210, j}}}, ~~~ j = 1, 2, 3, $$

where *z*_1−*α*/2_=1.96 is the 1−*α*/2 percentile of the standard normal distribution.

#### Bias and efficiency of the estimation methods

To compare the performance of the SHE, PTE and MLE, a simulation was performed to calculate bias, root mean square error (RMSE), length of 95 % CIs and coverage probabilities. We considered four different scenarios for the true proportion of successes at the end of treatment:

- Scenario 1: all treatments unpromising
  *p*_1_=*p*_2_=*p*_3_=0.75
- Scenario 2: all treatments promising
  *p*_1_=*p*_2_=*p*_3_=0.9
- Scenario 3: one treatment promising
  *p*_1_=*p*_2_=0.75, *p*_3_=0.9
- Scenario 4: linear relationship between efficacy and treatment
  *p*_1_=0.75, *p*_2_=0.825, *p*_3_=0.9

and four different cases for the change in patient status between the end of treatment and follow-up:

- Case 1: no relapses, no slow responders
  *q*=1, *s*=0
- Case 2: no relapses, 33 % slow responders
  *q*=1, *s*=0.33
- Case 3: 25 % relapses, no slow responders
  *q*=0.75, *s*=0
- Case 4: 25 % relapses, 33 % slow responders
  *q*=0.75, *s*=0.33

where *q* and *s* were defined as for the PTE. For each combination of the above, 10,000 simulations of Design 2 were performed.

We calculated the bias and RMSE of the three estimators after selecting the best performing treatment of each simulation. The best performing treatment refers to the treatment with the largest estimated success probability at follow-up. The bias and RMSE from selecting the best treatment were calculated using the following formulas:

$$\begin{array}{*{20}l} \text{bias} &= b_{p}(Q_{S}) = \mathrm{E_{p}}(Q_{S} - p'_{S}) \\ \end{array} $$

and

$$\begin{array}{*{20}l} \text{RMSE}_{p}(Q_{S}) &= \sqrt{\text{MSE}_{p}(Q_{S})} = \mathrm{\sqrt{E_{p}}\left[\left(Q_{S} - p'_{S}\right)^{2}\right]}\\ &= \sqrt{\text{Var}_{p}(Q_{p}) + {b^{2}_{p}}(Q_{S})}, \end{array} $$

where *S*∈(1,2,3) is the index of the selected treatment, *Q*_*S*_ is the estimator used and *p*^′^ is the true value of the efficacy at follow-up.

The length of the 95 % CIs is given as the upper confidence limit minus the lower confidence limit. The coverage probability of a CI gives the proportion of times the true value of the parameter (*p*^′^) lies within the interval. For a 95 % CI, we would, therefore, expect the coverage probability to be approximately 0.95.

### Trial registration and ethical approval

Ethical approval for the LEAP 0208 trial was obtained from the following committees: in Sudan, of the Institute of Endemic Diseases, University of Khartoum (reference IEND:UKIENDERC 2/11), and the Health Research Council, National Research Ethics Review Committee (reference 113-11-09), Ministry of Health of Sudan; in Kenya, of the Kenya Medical Research Institute (KEMRI EC, protocol 1720); and in the United Kingdom, of the London School of Hygiene and Tropical Medicine (reference 5543). The trial is registered with ClinicalTrials.gov, number NCT01067443.

## Results

### Fixing an interim analysis after a pre-specified number of responses

**Fig. 3:**
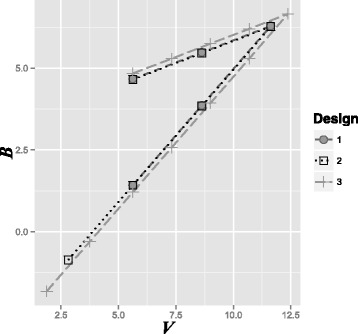
Three flexible triangular design boundaries. Boundaries for plotting the efficient score (*B*_*i*_) versus Fisher’s information (*V*_*i*_) for the three potential designs for the motivating example with a two-sided Type I error rate of *α*=0.05 and power of 0.95 when *θ*_R_=1.10

Figure 3 shows the three potential designs for the motivating example. One can see that the boundaries become wider and the maximum sample size becomes larger as the number of interim analyses is increased. The results of the simulation exercise are summarized in Table 1. The expected sample size decreases as the number of interim analyses increases. Although all three designs satisfied the Type I error of 5 %, a power of 95 % was not achieved, with the average being approximately 90 %. This reduction is due to the use of the normal approximation, which has limited accuracy when used with a single stream of binary data and when used with success probabilities close to 1 (see p. 235 [10]). A simple correction is to increase the sample size until the simulated power exceeds 95 % (see Table 2). More accurate approaches are available that take explicit account of the binomial distribution of the data [18, 19].

**Table 1:** Parameter estimates for the three potential designs for the motivating example. A simulation was performed to calculate expected sample size, Type I error and power

Estimate	Design 1	Design 2	Design 3
	(3 analyses)	(4 analyses)	(7 analyses)
Maximum sample size	62	62	66
Expected sample size under *H*_0_	36	31	30
Expected sample size under *H*_1_	40	40	39
Type I error	0.0479	0.0482	0.0484
Power	0.893	0.889	0.894

**Table 2:** Parameter estimates for the three potential designs for the motivating example under the maximum sample size required to satisfy power. A simulation was performed to calculate expected sample size, Type I error and power

Estimate	Design 1	Design 2	Design 3
	(3 analyses)	(4 analyses)	(7 analyses)
Maximum sample size	86	86	102
Expected sample size under *H*_0_	46	44	43
Expected sample size under *H*_1_	49	49	48
Type I error	0.0471	0.0453	0.044
Power	0.962	0.964	0.976

### Estimation of the probability of cure after extended follow-up

**Fig. 4:**
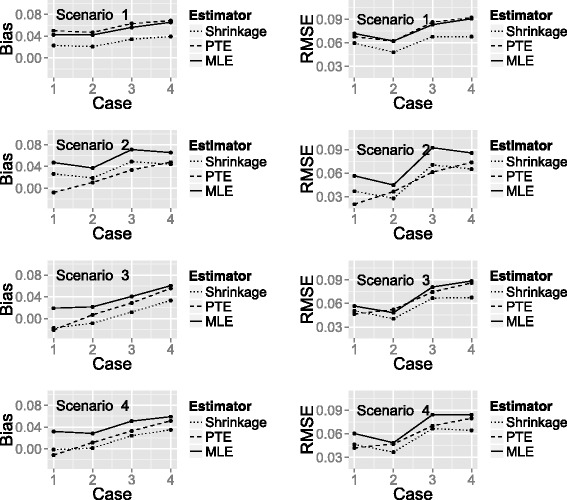
Plot showing the bias and RMSE of the three estimators. A plot of bias and RMSE for the SHE, PTE and MLE calculated under each true success probability and change in patient status combination

Figure 4 shows a plot of the bias and RMSE of the three estimators (SHE, PTE and MLE) calculated under each combination of the true success probability scenarios and change in patient status cases, with the latter represented on the *x*-axis. One can see that for all of the estimators, the bias and RMSE are higher for some scenarios than others. To some extent, this is explained by the closeness of the true probability to 1, which places an upper limit on the degree of the bias.

When all treatments are unpromising (Scenario 1), the SHE has the smallest bias and RMSE regardless of the change in patient status. In Scenario 2 (all treatments promising), the PTE has the smallest bias in all change of patient status cases. However, the performance of the estimators for RMSE differs dependent on the change in patient status. When there are no slow responders (patients not cured at end of treatment who become cured at extended follow-up), the PTE has the smallest RMSE; when there are slow responders, the SHE has the smallest RMSE.

When only one treatment is promising (Scenario 3) or when there is a linear relationship between efficacy and treatment (Scenario 4), the SHE has the smallest bias and RMSE in the majority of change in patient status cases. The PTE marginally outperforms the SHE when there is no change in patient status between the two time points.

**Fig. 5:**
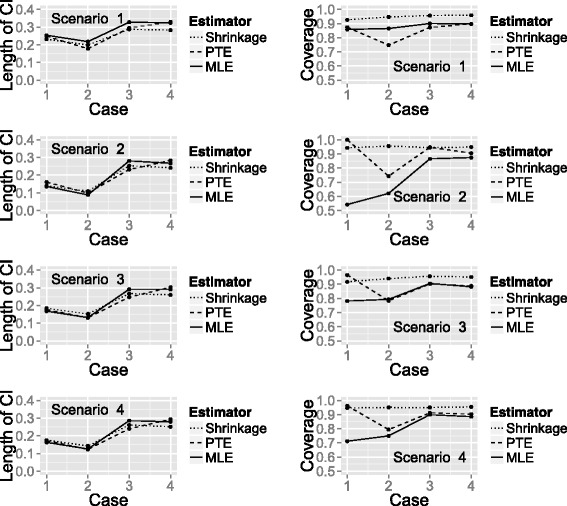
Plot showing the length of 95 % CIs and coverage probability of the three estimators. A plot of the length of 95 % CIs and coverage probability for the SHE, PTE and MLE calculated under each true success probability and change in patient status combination

Figure 5 shows the length of 95 % CIs and the coverage probability for the three estimators calculated under each combination of the true success probability scenarios and change in patient status cases, with the latter again represented on the *x*-axis. The length of the CIs is similar for all estimators whilst the coverage probability differs considerably between them. When all treatments are unpromising (Scenario 1), the SHE has much better coverage probability regardless of the change in patient status. In all other success probability scenarios, the SHE has the largest coverage probability in most cases, the exception being when there is no change in patient status between time points (Case 1) when the PTE performs better.

## Discussion

### Fixing an interim analysis after a pre-specified number of responses

The triangular test is a popular choice of sequential test due to its low expected sample size across a wide range of potential treatment effect sizes, and also for historical reasons, as very good approximations could be used to generate stopping boundaries without a computer. We have shown how one can modify the triangular test to fix an interim analysis after a pre-specified number of patients. This enables a trial designer to guarantee the minimum number of patients necessary to obtain sufficient PK data unless a treatment is unpromising.

Just as for the standard triangular test, it remains true that the effect of increasing the number of interim analyses is to reduce the expected sample size, but at the price of increasing the maximum total sample size (see Table 1). A greater number of interim analyses provides more opportunities to stop a trial early; however, it could make the trial less efficient to perform and increases the possibility of overrunning—data accumulated after the formal stopping criterion has been reached (see Section 5.5 [6]).

Stopping boundaries and sample sizes can be found to match specified Type I and Type II error rates. For normally distributed data, the error rates are achieved exactly. For many types of non-normal data [6], they are achieved approximately. The adequacy of this approximation can be assessed via simulation. In our setting, we found that a Type I error of 5 % was achieved; however, a power of 95 % used in the trial designs was not achieved and the average expected power was approximately 90 % (see Table 1). This reduction in power is due to the normal approximation performing poorly with success probabilities close to 1 (see p. 235 [10]).

In this phase II trial, the emphasis was on learning rather than confirming, and therefore no formal correction was made for the multiple hypotheses tested.

### Estimation of the probability of cure after extended follow-up

The second aim of the paper was to derive methods for estimation of the outcome variable (e.g., cure) at a time point later than that used for the stopping rule. In a trial for a new treatment approach for Indian VL [20], the impact of the group-sequential design was not considered in the analysis at the extended follow-up. Instead the MLE was used, which we know to be a biased estimator.

Simulation suggests that a SHE based on a Bayesian probit model is the preferred choice of estimator (out of the SHE, PTE and MLE) for estimation of the probability of cure after extended follow-up. The simulation was performed under a range of true success probability scenarios and change in patient status cases (it is possible for there to be slow responders to treatment or relapses). The SHE performed best in most situations in terms of reducing both bias and RMSE and providing coverage probabilities close to 0.95. The PTE performed well in instances where there was no change in patient status between end of treatment and follow-up. The MLE was poor in all instances. It is well known that for binary data, the MLE CI based on the normal approximation only performs well for large *n*. Agresti states that “the actual coverage probability usually falls below the nominal confidence coefficient, much below when *π* is near to 0 or 1”, where *π* is the true success proportion [21]. We would not recommend using the MLE for estimation of efficacy at follow-up based on these findings in any of the scenarios considered.

Whilst the SHE performs best, it is difficult to implement in comparison to the PTE and MLE. In particular, choosing the prior distribution for the variance random effects is a subtle task. The SHE would also be unsuitable for use in trials of a single treatment arm and so the PTE may provide a suitable alternative.

## Conclusions

Generalization of the triangular design is simple to implement and allows the minimum number of patients necessary for PK analysis to be obtained. For the estimation of efficacy at follow-up following a sequential design trial, a SHE is preferable. The PTE would provide an alternative for use in one-arm trials or when the SHE is not possible due to its computational complexity.

## Additional file

Additional file 1
**Supplementary R functions.** Zip file containing all R code necessary to reproduce our results, together with description files that explain how to use the functions. Installation instructions can be found at: http://cran.r-project.org/doc/manuals/r-release/R-admin.html#Installing-packages. (PDF 463 kb)

## Abbreviations

CI: confidence interval
MCMC: Markov chain Monte Carlo
MLE: maximum likelihood estimator
PK: pharmacokinetic
PTE: probability tree estimator
RMSE: root mean square error
SHE: shrinkage estimator
VL: visceral leishmaniasis
