# Supplementary material for: Generalizing boundaries for triangular designs, and efficacy estimation at extended follow-ups
Source: Trials. 2015 Nov 16;16:522. doi: 10.1186/s13063-015-1018-1 (PMC4647805; doi:10.1186/s13063-015-1018-1)
Supplement: Additional file 1 — Supplementary R functions. Zip file containing all R code necessary to reproduce our results, together with description files that explain how to use the functions. Installation instructions can be found at: http://cran.r-project.org/doc/manuals/r-release/R-admin.html#Installing-packages. (PDF 463 kb) [file 13063_2015_1018_MOESM1_ESM.zip › gentri/html/00Index.html]

R: generalizing triangular boundaries and efficacy estimation at
extended follow-up

# generalizing triangular boundaries and efficacy estimation at extended follow-up

---

## Documentation for package ‘gentri’ version 1.3

- DESCRIPTION file.

## Help Pages

|  |  |
| --- | --- |
| gentri-package | Generalized triangular tests and efficacy estimation after extended follow-up |
| bias\_mse\_coverage | Bias, mean-square-error and coverage |
| compare\_estimators | Compare the performance of mle, pte and she estimators |
| find\_design | Find stopping boundaries and sample size for a generalized triangular test |
| gentri | Generalized triangular tests and efficacy estimation after extended follow-up |
| mle\_210 | Maximum likelihood estimate of day 210 success probability and associated confidence interval |
| plot.gen\_tri | Generic functions for class gen\_tri |
| plot\_bias\_rmse | Plot comparison of bias and root-mean-square-error |
| plot\_coverage\_length | Plot comparison of average lengths and coverage probabilities of confidence/credible intervals |
| print.gen\_tri | Generic functions for class gen\_tri |
| probit\_gibbs | Gibbs sampler for a simple probit model |
| pte\_210 | Probability tree estimate of day 210 success probability and associated confidence interval |
| she\_210 | Shrinkage estimate of day 210 success probability and associated credible interval |
| sim\_28\_210 | Simulate success/failure responses after both 28 days of treatment and 210 days of follow-up |
